# The role of task demands in racial face encoding

**DOI:** 10.1038/s41598-022-19880-4

**Published:** 2022-11-07

**Authors:** Bo Yang, Jialin Ma, Ran Ding, Xinyi Xia, Xiaobing Ding

**Affiliations:** 1 Department of Psychology, Sun Yat-Sen University, Guangzhou, 511400 China; 2 Faculty of Education, Henan University, Kaifeng, 475000 China; 3 School of Psychology, Northwest Normal University, Lanzhou, 730000 China

**Keywords:** Human behaviour, Cognitive neuroscience

## Abstract

People more accurately remember faces of their own racial group compared to faces of other racial groups; this phenomenon is called the other-race effect. To date, numerous researchers have devoted themselves to exploring the reasons for this other-race effect, and they have posited several theoretical explanations. One integrated explanation is the categorization-individuation model, which addresses two primary ways (categorization and individuation) of racial face processing and emphasizes the emergence of these two ways during the encoding stage. Learning-recognition and racial categorization tasks are two classical tasks used to explore racial face processing. Event-related potentials can facilitate investigation of the encoding differences of own- and other-race faces under these two typical task demands. Unfortunately, to date, results have been mixed. In the current study, we investigated whether categorization and individuation differ for own- and other-race faces during the encoding stage by using racial categorization and learning-recognition tasks. We found that task demands not only influence the encoding of racial faces, but also have a more profound effect in the encoding stage of recognition tasks for other-race faces. More specifically, own-race faces demonstrate deeper structural encoding than other-race faces, with less attentional involvement. Moreover, recognitions tasks might ask for more individual-level encoding, requiring more attentional resources in the early stage that may be maintained until relatively late stages. Our results provide some evidence concerning task selection for future racial face studies and establish a groundwork for a unified interpretation of racial face encoding.

## Introduction

Humans are experts in facial recognition but can more accurately remember faces of their own race relative to faces of individuals of other races. This phenomenon has been called the other-race effect or own-race bias^1^. While individuals tend to better remember own-race faces, less time is needed to categorize faces of other races, an effect referred to as other-race category advantage^2–5^. Several theoretical explanations have been proposed to account for these phenomena, including perceptual experience theory^6^, the race-feature hypothesis^7,8^, the multidimensional space hypothesis^9^, and social cognitive theory^10^. Through integration of previous theories, Hugenberg and Young^11^ posited the categorization-individuation model (CIM) that there were two different ways of processing faces during the encoding stage: categorization and individuation. Categorization refers to the classification of the face into a certain group based on facial characteristics (e.g., skin tone). Individuation consists of distinguishing and identifying a face as belonging to a specific individual. Although categorization and individuation play an important role in shaping racial face encoding, the two ways do not have equal influences. Categorization, the first stage of facial encoding, occurs faster for other-race faces than for own-race faces, which causes observers to have less motivation to individualize other-race faces, whereas does not influence the automatic individuation of own-races faces. Consequently, other-race faces are classified by race faster, whereas own-race faces are more accurately recognized. Thus far, numerous studies have used various tasks to investigate differences in categorization and individuation of faces.

A variety of classical tasks have been used to investigate racial face processing, including the learning-recognition paradigm^12–14^, the oddball paradigm^15,16^, face orientation judgment^17^, face/race categorization task^2,18^, visual search task^8^, remember–know–guess judgment^19^, and the repetition lag paradigm^20^. For example, Levin^8^, using a visual search task, showed that faces of other races were more easily detected than faces of the individual’s own race. Moreover, using the learning-recognition paradigm, Wiese, Kaufmann, and Schweinberger^13^ asked participants to complete an additional racial categorization task in the learning phase and found that own-race faces were categorized more slowly, but were better recognized than other-race faces. Among the wide variety of research paradigms mentioned above, the racial categorization task and learning-recognition paradigm are highly favored in addressing racial face processing. By using these two tasks, researchers can directly compare the categorical and individual processing of racial faces.

Although, these studies provide some evidence that supports the categorization-individuation model (CIM), Hugenberg and Young^11^ pointed out that differences between categorization and individuation processing occur almost exclusively during facial encoding (rather than during storage or retrieval). Event-related potentials (ERPs) are an appropriate method to test individuation and categorization encoding of racial faces. Of the possible time points to measure ERPs, N170 has been shown to be the most important component in face related studies. N170 is a negative deflection that peaks at approximately 170 ms over occipito-temporal sites, and is thought to reflect the structural encoding of faces^21^ or configural processing of facial features^22^. Interestingly, results from previous studies at N170 under these two tasks are inconsistent. For racial categorization tasks, the majority of previous studies have found a non-significant difference in N170 of own- and other races^3,23–29^; while other studies have reported either a larger N170 for own- relative to other-race faces^30^, or a reversed trend, in which other-race faces exhibit a larger N170 when compared to own-race faces^2,5^. Previous results examining N170 during a learning-recognition task are even more complicated, as many studies embedded other tasks in the learning phase, such as a racial categorization task, or facial attractiveness evaluation task^12–14,27,31^. Although the result of N170 was reported in many of these studies, it was derived from different stages of the learning-recognition task, including the learning^12–14^ and test phases^27^. Ito and Senholzi^4^ suggested that these inconsistent results raised the question as to whether categorical and individuation processing of racial faces might be brought about by task demands, rather than racial face processing per se. Similarly, Wiese^27^ posited that task demands play an important role in the emergence of the N170 racial effect.

In this current study, we aimed to answer the simple and important question of whether categorization and individuation differ for processing of own- and other-race faces during the encoding stage. To address this aim, we used racial categorization and learning-recognition tasks, to provide information for future studies related to racial face encoding.

In addition to the N170 component, several other ERP components were of interest in this study. The early component, P1, which is a positive deflection over occipital areas, peaks at approximately 100 ms after the presentation of a visual stimulus. P1 is sensitive to basic stimulus properties, such as spatial frequency, contrast, and luminance^32^, but is also modulated by stimulus category^33^ and influenced by selective attention^34^. Recent studies have found P1 might also be modulated by task demands. Colombatto and McCarthy^35^, using a categorization task demand, found own-race faces had a larger P1 than other-race faces. As for demand of an individualization task, Wang and colleagues^36^ found P1 was influenced by race of faces and facial configural processing when face was attended to.

The midline P2 component is a positive deflection peaking approximately between 100–250 ms, which has been associated with selective attention^37^, and is considered an index of other racial-processing^38^. Research has shown larger P2 amplitudes for other-race faces, revealing greater attention orientation to other-race faces^15,16,26,38–44^, with a majority of these studies have using a racial categorization task demand. This larger amplitude of P2 for other-race faces appeared robust, even being observed when the categorization task was focused on gender^15,44,45^. Volpert-Esmond, et al.^46^ compared a race-irrelevant task (evaluation priming) and racial categorization task, and observed a larger P2 for Black than White faces from White participants for both tasks. However, Ito, et al.^47^ asked participants to passively look at racial faces, and think about whether they like or dislike the particular face being viewed, and did not observe a P2 difference in Black and White faces from White participants. At minimum, the results of these two studies suggest a potential influence of task demands on the amplitude of P2 for racial faces. Furthermore, when Asian faces are taken into consideration, results are also mixed^41^, Willadsen-Jensen and Ito^42^ found that both Asian and White participants have larger P2 for other-race faces using a racial categorization task; whereas, He, et al.^48^ did not observe a difference in P2 between White and Asian faces for White participants using a racial irrelevant task. Nevertheless, the CIM framework describing the two methods of encoding own-face and other-race faces was applied in the current study to directly compare how individualization and categorization task demands affect P2 when encoding racial faces, so that we could explore whether attentional involvement of P2 results from differences in processing quantity of facial categories and whether task demands also play a role in this processing for Asian participants.

P300 is the third ERP component to be investigated in our study. It is the most positive peak between 300 and 600 ms and is particularly sensitive to social category information^15,39^, task complexity^49^, degree of relevance, and motivational significance of stimuli^50^. Specifically, P300 amplitude varies between different task demands, reflecting focused attention on facial processing^51^ and discrimination between differences in identity^52^. While some studies related to racial faces have observed larger P300 amplitude for other-race faces when compared to own-race faces in racial categorization tasks^28,29^, another study, utilizing an identity matching task did not observe racial differences in P300^36^. However, despite the White and Asian faces used in these three studies, the racial results of P300 still varied between task demands. In the current study, we hoped to provide evidence of this important component related to racial face processing under recognition and racial categorization tasks at the encoding stage.

The present study aimed to investigate the effect of task demand on neural processing of own- and other-race faces during the encoding stage. By comparing the categorization task with the learning phase of the recognition task, based on the CIM, we hypothesized that task demands would not automatically affect the individual processing of own-race faces. Thus, own-race faces should demonstrate similar N170 for both categorization and recognition tasks, whereas N170 would be larger for recognition tasks for other-race faces, when compared to categorization tasks. Since we were uncertain how the task demands influenced racial face encoding related to P1, P2, and P300, we had no strong predictions regarding such conditions.

## Methods

### Participants

Twenty-seven Chinese participants (13 male, mean age 24.13 ± 2.00 SD) were initially selected. Ethical approval for the study was granted by Northwest Normal University in accordance with the Declaration of Helsinki. All participants signed informed consent before the experiment.

### Stimuli

Stimuli consisted of 100 White (50 female) and 100 Asian (50 female) faces with neutral expressions that were selected from the CAL/PAL Database^53^. All faces were converted to grayscale, and placed on a black background (240 × 240 pixels), informed consent to publish identifying images was obtained.

### Procedure

Throughout the experiment, participants sat in a stationary chair in a dimly lit room, holding a distance of approximately 60 cm. A 23-inch monitor with a resolution of 1920 × 1080 pixels was used for the experiments. Before the formal experiment, participants completed 8 practice trials to familiarize themselves with the experimental procedure and response button. Each participant completed eight task demand blocks (four blocks of categorization tasks and four blocks of recognition tasks) in the formal experiment, which were counterbalanced across participants (e.g., ABABABAB and BABABABA). There were 128 faces randomly assigned to four recognition task blocks, with 32 faces (half female, half Asian) in each block. Another different 64 faces were used in four categorization task blocks with 16 faces of each (half female, half Asian).

**Figure 1:**
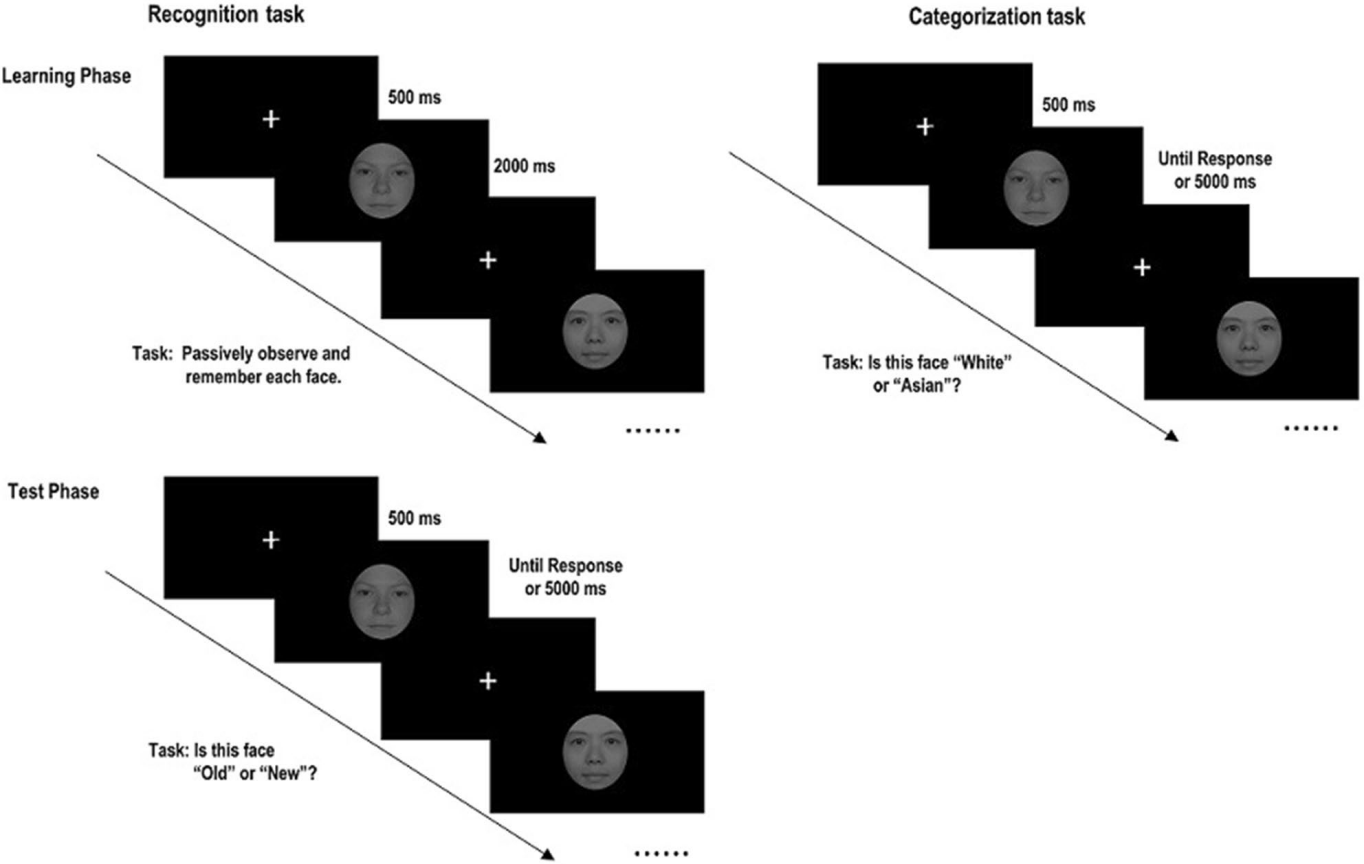
Experimental procedure of recognition and racial categorization tasks.

Each recognition task block included a learning and a test phase. During the learning phase, 16 faces (half female, half Asian) were presented randomly once, and then learned again in the same order. Participants were asked to remember the presented faces. Each trial started with a fixation (500 ms), followed by presentation of the face (2000 ms) that participants were asked to passively observe and remember. The test phase started when participants indicated they were ready. In the test phase, 32 faces were presented randomly (16 previously presented faces and 16 new faces). In the test phase, after the fixation (500 ms), the test face was presented for a maximum duration of 5000 ms or when the participant made a response. Participants were asked to press "F" for previously-viewed faces or "J" for new faces; then, the next trial was started (see Fig. 1).

For each categorization block, 16 faces were randomly presented. Participants were asked to determine whether the presented face was White or Asian as accurately and quickly as possible. Each trial started with a fixation (500 ms), then a racial face was displayed for a maximum of 5000 ms, or until the participant pressed a response key: "F" for White faces and "J" for Asian faces (counterbalanced across participants) (see Fig. 1).

### EEG recording and analysis

EEG was recorded using 64 Ag/AgCl electrodes mounted on an elastic cap (eego sports, ANT Neuro, Inc) and continuously sampled at 500 Hz by an AsaLab Amplifier (www.ant-neuro.com), which was referenced on-line to the vertical central parietal (Cpz). All electrode impedances were kept below 5 KΩ. EEG data processing was performed off-line using EEGLab, recalculated to average reference, and band-pass filtered (0.1–30 Hz). Contributions of eye blink artifacts were corrected. The EEG was segmented from − 200 to 800 ms relative to stimulus onset, with the first − 200 ms as baseline. To eliminate artifacts, we used an independent component analysis (ICA) from the EEGLAB toolbox^54^ with *ICLabel*^55^ to distinguish and exclude non-brain components. The remaining artifacts exceeding ± 100 mV amplitude were rejected. After preprocessing, the mean number of remaining trials were 110.0 and 108.9 (*SD* = 25.0, 25.0) for White and Asian faces of the learning phase of the recognition task, respectively, and 47.6 and 47.8 (*SD* = 17.1, 17.5) in the categorization task, respectively. Electrode selection was based on previous studies^28,30,31^. The peak latency of each component was selected based on the grand average waveform across all participants and conditions (as recommended by Luck and Kappenman^56^). Then, a ± 20 ms-window around the grand average peak latency for P1 and N170, ± 30 ms-window for P2, and a ± 200 ms-window for P300, were used to calculate the mean amplitude of each respective measure^56^, with P1 (120–160 ms O1/O2^24^), N170 (180–220 ms P7/P8/PO7/PO8^57^), P2 (180–240 ms Fz/ Cz/ Pz^26,41^), and P300 (400–800 ms Fz, Cz, Pz, Oz^28^). Since the main purpose was to compare the perceptual differences of racial faces under the two different task demands, we only recorded the learning phase of the recognition task to enable direct comparison with the categorization task to be made. Greenhouse–Geisser corrections were used to address violations of sphericity. A Bonferroni correction was applied for each pairwise comparison of all experiments in this study to control for type I error.

## Results

### Behavioral results

The mean corrected reaction times (RT) of the two face types under the recognition task demand (White: 1046.90 ± 212.25 ms, Asian: 1069.72 ± 260.71 ms) the and categorization task demand (White: 779.29 ± 154.98 ms, Asian: 820.70 ± 167.63 ms) were analyzed by a repeated-measures ANOVA, with race (White vs. Asian) and task demands (recognition vs. categorization) as within-subject factors. The main effect of task demands was significant, *F*(1, 26) = 90.536, *p* < 0.001, η_*p*_^*2*^ = 0.777, with a faster response speed in the categorization task than the recognition task. The main effect of race was also significant, *F*(1, 26) = 6.989, *p* = 0.014, η_*p*_^*2*^ = 0.212, with White faces exhibiting faster response speed when compared to Asian faces. However, the interaction between task demands and face race was not significant (*p* = 0.524, η^*2*^_*p*_ = 0.016).

We analyzed accuracy of race under the recognition (White: 0.75 ± 0.07, Asian: 0.74 ± 0.09) and categorization (White: 0.93 ± 0.07, Asian: 0.94 ± 0.07) task demands using the same analysis method of RT. The main effect of task demand was significant, *F*(1, 26) = 117.005, *p* < 0.001, η_*p*_^*2*^ = 0.818, reflecting more accuracy in the categorization task relative to the recognition task. Both the main effect of race and the interaction between task demands and race were not significant (*p*s > 0.338, ηs^*2*^_*p*_ < 0.035).

### EEG results

**Figure 2:**
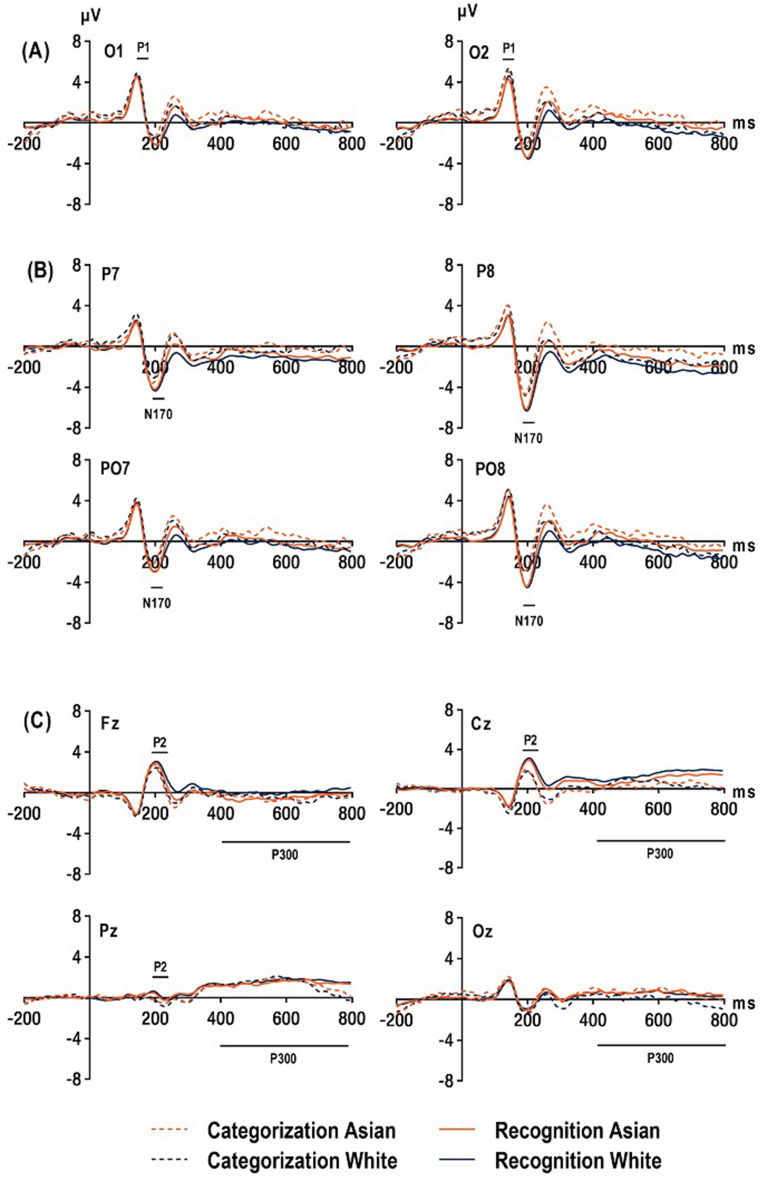
Grand average waveforms. (**A**) P1 at O1/O2 in the 120–160 ms time window, (**B**) N170 at P7/8 and PO7/8 in the 180–220 ms time window, (**C**) P2 at Fz/ Cz/ Pz in the 180–240 ms time window, P300 at Fz, Cz, Pz, Oz in the 400–800 ms time window.

For P1, a 2 (race: White, Asian) × 2 (task demand: recognition, categorization) × 2 (hemisphere: O1, O2) repeated-measure ANOVA was conducted (see Fig. 2A). The main effect of task demand was significant, *F*(1, 26) = 9.926, *p* = 0.004, η^*2*^_*p*_ = 0.276, with the categorization task (1.56 ± 0.48 µV) exhibiting a larger amplitude than the recognition task. The interaction between task demand and hemisphere was also significant *F*(1, 26) = 10.381, *p* = 0.003, η^*2*^_*p*_ = 0.285, with the categorization task demonstrating a larger amplitude than the recognition task in both the left (1.24 ± 0.48 vs. 0.59 ± 0.40; *p* = 0.039) and right (1.88 ± 0.52 vs. 0.68 ± 0.41 µV; *p* < 0.001) hemispheres, but the variations between the two task-evoked P1 amplitudes were different, with a larger difference noted in the left hemisphere. Other main effects and the interaction did not reach significant levels (*p*s > 0.174, ηs^*2*^_*p*_ < 0.070).

For N170, a 2 (race: White, Asian) × 2 (task demand: recognition, categorization) × 2 (hemisphere) × 2 (site: P7/8, PO7/8) repeated-measure ANOVA was performed (see Fig. 2B). The main effect of task demand for N170 was significant *F*(1, 26) = 14.786, *p* = 0.001, η^*2*^_*p*_ = 0.363, with recognition (− 3.95 ± 0.64 µV) demonstrating a larger N170 amplitude than categorization (− 2.65 ± 0.56 µV) task demand. The main effect of race and the interaction effect of race and task demand were not significant (*p*s > 0.215, ηs^*2*^_*p*_ < 0.059). The interaction between the hemisphere and task demand was significant *F*(1, 26) = 8.856, *p* = 0.006, η^*2*^_*p*_ = 0.254; further analysis found that the right hemisphere demonstrated larger N170 amplitudes than the left hemisphere for both categorization (− 3.06 ± 0.66 vs. − 2.23 ± 0.50; *p* = 0.024) and recognition tasks (− 4.44 ± 0.75 vs. − 3.24 ± 0.57; *p* = 0.001), but with differing magnitudes. The interaction between hemisphere and race was significant *F*(1, 26) = 8.225, *p* = 0.008, η^*2*^_*p*_ = 0.240, with White faces (− 4.02 ± 0.67 µV) evoking larger N170 amplitude than Asian faces (− 3.79 ± 0.69 µV) in the right hemisphere (*p* = 0.022, η^*2*^_*p*_ = 0.186), and the N170 amplitude of White faces (− 2.71 ± 0.52 µV) and Asian faces (− 2.76 ± 0.51 µV) was not significantly different at the left hemisphere (*p* = 0.713, η^*2*^_*p*_ = 0.005). The interaction between site, task demand, and race was significant *F*(1, 26) = 8.016, *p* = 0.009, η^*2*^_*p*_ = 0.236. Further separate analysis found that the interaction between site and race was significant for the recognition task *F*(1, 26) = 4.400, *p* = 0.046, η^*2*^_*p*_ = 0.145, with a marginally significantly larger N170 amplitude for White (− 4.81 ± 0.66 µV) than Asian (− 4.55 ± 0.68 µV) faces at P7/8 (*p* = 0.090), whereas a non-significant difference of N170 amplitude was found for both White (− 3.16 ± 0.64 µV) and Asian (− 3.28 ± 0.63 µV) faces at PO7/8 (*p* = 0.458). The interaction between the site and race was not significant in the categorization task *F*(1, 26) = 3.067, *p* = 0.092, η^*2*^_*p*_ = 0.106. Other post hoc separation analyses of site, task and race did not exhibit significant two-way interaction effects (*ps* > 0.147, ηs^*2*^_*p*_ < 0.079). The interaction between task demand, race, and hemisphere was not significant *F*(1, 26) = 2.510, *p* = 0.125, η^*2*^_*p*_ = 0.088, for the left hemisphere (P/PO7), the interaction between task demand and race was not significant *F*(1, 26) = 0.900, *p* = 0.352, η^*2*^_*p*_ = 0.033. Regarding the right hemisphere (P/PO8), the interaction between task demand and race did not reach a significant level *F*(1, 26) = 0.153, *p* = 0.69, η^*2*^_*p*_ = 0.006. The main effect of site was significant *F*(1, 26) = 39.848, *p* < 0.001, η^*2*^_*p*_ = 0.605, with P7/8 (− 4.01 ± 0.60 µV) demonstrating a larger N170 amplitude than PO7/8 (− 2.58 ± 0.57 µV). The main effect of the hemisphere was significant *F*(1, 26) = 10.036, *p* = 0.004, η^*2*^_*p*_ = 0.279, so larger N170 amplitudes were observed for the right (− 3.86 ± 0.68 µV) versus (− 2.74 ± 0.51 µV) the left hemisphere. The other main and interaction effects were not significant (*p*s > 0.071, ηs^*2*^_*p*_ < 0.120).

Analysis of P2 was conducted with a 2 (race: White, Asian) × 2 (task demand: recognition, categorization) × 3 (site: Fz, Cz, Pz) repeated-measured ANOVA (see Fig. 2C). A significant main effect for task demand was observed, *F*(1, 26) = 31.402, *p* < 0.001, η^*2*^_*p*_ = 0.547, with the recognition (1.62 ± 0.27 µV) task exhibiting a larger P2 amplitude than the categorization (0.77 ± 0.24 µV) task. The main effect of race was significant, *F*(1, 26) = 4.377, *p* = 0.046, η^*2*^_*p*_ = 0.144, with White faces (1.26 ± 0.25 µV) resulting in a larger P2 than Asian(1.13 ± 0.24 µV) faces. The main effect of site was also significant, *F*(2, 52) = 15.132, *p* < 0.001, η^*2*^_*p*_ = 0.368, reflecting a larger amplitude of P2 at Fz than Pz (1.96 ± 0.36 vs. 1.72 ± 0.38 µV; *p* = 0.001), and Cz than Pz (1.72 ± 0.38 vs. − 0.101 ± 0.26 µV; *p* < 0.001). No other main or interaction effects reached significance (*p*s > 0.057, ηs^*2*^_*p*_ < 0.132).

For P300, a 2 (race: White, Asian) × 2 (task demand: recognition, categorization) × 4 (site: Fz, Cz, Pz, Oz) repeated-measured ANOVA was performed (see Fig. 2C). The main effect of task demand was significant *F*(1, 26) = 16.538, *p* < 0.001, η^*2*^_*p*_ = 0.389, revealing that the recognition task (0.81 ± 0.15 µV) demonstrated a larger P300 than the categorization task (0.39 ± 0.20 µV). The main effect of site was significant *F*(3, 78) = 4.071, *p* = 0.027, η^*2*^_*p*_ = 0.135, reflecting a larger amplitude of P300 at Pz (1.41 ± 0.32 µV) than Fz (− 0.32 ± 0.33 µV; *p* = 0.024) and Cz (0.84 ± 0.26 µV; *p* = 0.035). The interaction between the site and race was significant, *F*(3, 78) = 6.610, *p* = 0.003, η^*2*^_*p*_ = 0.203. Post-hoc analysis found that for Asian faces, a smaller amplitude of P300 was found at Fz (− 0.47 ± 0.34 µV) than Cz (0.69 ± 0.29 µV; *p* = 0.036) and Pz (1.34 ± 0.31 µV; *p* = 0.018); for White faces, a smaller amplitude of P300 was found at Fz (− 0.18 ± 0.33 µV) than Cz (0.97 ± 0.25 µV; *p* = 0.020). The other main and interaction effects were not significant (*p*s > 0.100, ηs^*2*^_*p*_ < 0.101).

To confirm that the given number of participants in the study were allowed to detect of sufficient power, we conducted a sensitivity power analysis using G*Power software^58^. The sensitivity power analysis can assess the minimal effect size to achieve 80% power when the sample size and alpha level are fixed^59^. A repeated measurement of ANOVA Analysis with 27 participants in our study would be sensitive to the effects of *f* = 0.230 (η^2^_*p*_ = 0.050) with 80% power (α = 0.05), revealing that an observed effect size larger than η^2^_*p*_ = 0.050 would have 80% power to detect the effect.

## Discussion

Using a within-subject design, the present study investigated the effect of task demand on neural processing of own- and other-race faces during the encoding stage. The behavioral results demonstrate that other-race faces had a faster categorization speed than own-race faces. With regard to ERP results, we observed a larger P1 for the categorization task than the learning phase of the recognition task. Conversely, N170 and P2 demonstrated a larger amplitude for the learning phase of the recognition task than the categorization task. Moreover, both N170 and P2 revealed larger amplitudes for other relative to own-race faces, but this result of N170 amplitude was only observed at the right hemisphere. Specifically, for N170, a marginally enhanced N170 for other-race faces was observed during learning of the recognition task at anterior locations (P7/8). Finally, P300 was larger for recognition relative to racial categorization task. These ERP results are discussed in the following paragraphs.

The P1 analysis revealed that P1 was larger for the categorization task than the learning phase of the recognition task. Although previous studies have suggested that the P1 component is sensitive to visual properties, such as luminance^60,61^ and contrast^32^, the main effect of enhanced P1 for the categorization task compared to the learning phase of the recognition task is unlikely due to the variation of these low-level differences, since the racial faces were randomly assigned across two tasks, and the presentation of the two tasks was counterbalanced across participants. Stahl, et al.^12^, using a recognition task, and with additional instruction in the learning phase that either asked participants to complete a attractiveness rating task or a racial categorization task, found that the categorization task demonstrated a larger P1 than the attractiveness rating task. Similarly, such differences between task demands of P1 in our experiment also reflected differences of early attentional arousal in early visual processing of different task demands.

We found that other-race faces elicited larger N170 amplitudes than own-race faces at the right hemisphere. The racial effect was consistent with that of previous studies^2,5,13^. However, if we narrow down the facial race stimuli to White and Asian and the race of participants to Asian and White, the majority of studies did not observe that the racial effect was on N170^3,23–29^, and only Wiese et al.^13^ found a larger N170 for other-race faces in the learning phase. Note that in this study, the learning phases of the recognition task embedded an additional racial categorization task. Indeed, participants were invited to complete two different racial tasks, similar to our study. It seems that when participants were asked to process racial faces that simultaneously included racial categorization and individualization demands, a larger N170 for other than own-race faces would be observed. The possible explanation is that the simultaneous completion of categorization and individuation demands requires the processing of both racial category and face identity information, leading to deeper encoding than the single-task demand, thus causing differences between the racial faces to emerge. Considering the decreased amplitude of N170 reflects deeper structural encoding of faces^21^, such as upright compared to inverted faces^21,62^. Therefore, the increased N170 amplitudes of other-race faces observed in the current study reflect that the structural processing of own-race faces is stronger than other-race faces for Chinese participants at the right hemisphere.

Additionally, we found larger N170 amplitude in the learning phase of the recognition task compared to categorization task. The task demand of recognition typically demonstrates deeper overall individual encoding when compared to the racial categorization. This might be interpreted as the memorization instruction in the recognition task inducing individual processing^12^ of racial faces, resulting in deeper individual face encoding in the recognition task compared to race categorization task. Therefore, our result indicates that the task demands alone could induce the differences observed in N170, which may enhance the individual identity processing of racial faces.

Most importantly, we observed an interaction between site, facial race, and task demand. Marginal differences between own- and other-race faces at P7/8 were observed for the recognition task. Although this racial effect only reached marginal significance in our study, the trend of observing the racial effect of N170 at anterior electrodes for the recognition task is consistent with that of Wiese, et al.^13^. This trend suggested that when the task demand asked the participant to perform individual-level encoding of racial faces, own-race faces probably elicited stronger structural encoding than other-race faces, which is not in line with the current CIM-based hypothesis. In fact, this result does not appear to be explained by other theories, such as perceptual experience theory^6^ or social cognitive theory^10^. Thus, the theory that accounts for these differences remains to be elucidated.

The current results show that other-race faces elicited a larger P2 than own-race faces, which is consistent with the results of the majority of previous studies^15,16,26,38–42,42–44^. Considering that smaller amplitude of P2 reflects less attentional orientation to racial faces^40^, and Willadsen-Jensen and Ito^41,42^ found the consistent result that both Asian and White participants had a larger P2 for other-race faces, we can infer that the overall encoding of own-race faces employs less attention than other-race faces for Chinese participants. Additionally, we observed that larger P2 amplitudes for recognition task compared to the racial categorization task. P2 has been shown to be sensitive to racial categorization information, with the enhancement of P2 reflecting increased attentional involvement^40,45^. This result indicates that the recognition task may require enhanced attention allocation compared to the categorization task. Furthermore, in previous studies^41^,Willadsen-Jensen and Ito^42^ observed a larger P2 for other-race faces using a racial categorization task for both Asian and White participants. However, He, et al.^48^ did not observe a difference in P2 between White and Asian faces for White participants. This inconsistency might be accounted by the task demand, as they asked participants to complete a racially irrelevant task (gender categorization). By using two racial related tasks, racial categorization and recognition task demands, we still observed that other-race faces elicited a larger P2 than own-race faces. Thus, we can infer that this race-related P2 component has inter-task stability, which can help us understand the attention resource allocation of racial face encoding in further studies.

In addition, when comparing the results of N170 and P2 together, we found some interesting patterns. Overall, other-race faces elicited larger amplitudes than own-race faces of N170 (at the right hemisphere) and P2 (at both hemispheres). Furthermore, both N170 and P2 observed larger amplitudes for the recognition task relative to categorization task. These similar trends of results of N170 and P2 raise an alternative explanation of P2; that is, P2 may share similar cognitive facial processes as N170^45^, which may be the so-called vertex positive potential (VPP)^63^. Meanwhile, the analyzed electrodes of P2 in our study overlapped with the electrodes’ sites of VPP (peaking at fronto-central sites). Just as Volpert-Esmond and Bartholow^45^ posited that the studies related to P2 may be integrated with VPP. In our study, the midline P2 component may alternatively be a measurement of VPP. So far, the facial-related P2 and VPP have not been integrated yet, but our results provide some evidence that can be used in future studies.

As for P300, the recognition task elicited an enhanced amplitude of P300 when compared to the categorization task. In previous studies, P300 was influenced by the task complexity^49^, and discrimination between differences in identity^52^, social category information^15,39^, which require less cognitive resources, resulted in a lower amplitude of P300^64^. Therefore, in our experiment, the larger P300 of the recognition task may demonstrate an increase in the cognitive resources needed when compared to the racial categorization task. Furthermore, a meta-analysis by Meissner and Brigham^65^ found that recognition tasks have similar processing mechanisms to perceptual individualization tasks, with the instruction to memorize faces given in the recognition paradigm possibly constituting individualization^12^. Hence, one possible inference is that the individualization processing underlying the recognition task requires more cognitive resources than categorization of racial face. However, considering that the significance of P300 might be a consequence of the difference found for P2, we should be cautious about this conclusion.

As only Chinese participants were involved in our study, we could not estimate how the experience affected our results. To thoroughly investigate the question that we posited, an ideal study design should be a complete cross-over design (i.e., both Asian and White participants). In addition, although the sensitivity power analysis provided evidence that supported the robustness of the results in the current study, there still existed the possibility that the overall power is underpowered because of the small sample size^66^. A study with overall low power would reduce the chance of detecting the true effect and the reproducibility of significant results^66^. Further studies should adopt larger sample size to increase the possibility of detecting a true effect^66^, to ensure that the effect size would not be inflated^66,67^, and to avoid false-positive results^68^. Besides, O'Toole et al.^69^ suggested that race typicality is related to racial face memorization. In future studies, using more other-race faces and adopting a cross-over design of participants may help reveal clearer evidence relating to how memory goals and task demand play a role in racial face processing.

## Conclusions

The present results provide evidence that task demands not only influence the encoding of racial faces, but also has a more profound effect on the encoding stage of a recognition task for other-race faces when compared to own-race faces. More specifically, own-race faces elicit deeper structural encoding than other-race faces (N170), with less attentional involvement (P2). Moreover, a recognition task might promote individual-level encoding (N170) requiring more attentional resources in the early (P2) stage that are potentially maintained until relatively late (P300) stages. These results may assist researchers in making clearer inferences in future racial face studies.

## Data Availability

The data generated during and/or analyzed during the current study are available on the Open Science Framework repository, https://osf.io/5skxe/.

## References

[1] Malpass RS, Kravitz J (1969). Recognition for faces of own and other race. J. Pers. Soc. Psychol..

[2] Caharel S (2011). Other-race and inversion effects during the structural encoding stage of face processing in a race categorization task: an event-related brain potential study. Int. J. Psychophysiol..

[3] Caldara R, Rossion B, Bovet P, Hauert CA (2004). Event-related potentials and time course of the ‘other-race’ face classi¢cation advantage. NeuroReport.

[4] Ito TA, Senholzi KB (2013). Us versus them: Understanding the process of race perception with event-related brain potentials. Vis. Cogn..

[5] Montalan B (2013). Investigation of effects of face rotation on race processing: An ERPs study. Brain Cogn..

[6] Rhodes G, Brake S, Taylor K, Tan S (1989). Expertise and configural coding in face recognition. Br. J. Psychol..

[7] Levin DT (1996). Classifying faces by race: The structure of face categories. J. Exp. Psychol. Learn. Mem. Cogn..

[8] Levin DT (2000). Race as a visual feature: Using visual search and perceptual discrimination tasks to understand face categories and the cross-race recognition deficit. J. Exp. Psychol. Gen..

[9] Valentine T, Endo M (1992). Towards an exemplar model of face processing: The effects of race and distinctiveness. Q. J. Exp. Psychol..

[10] Sporer SL (2001). Recognizing faces of other ethnic groups: An integration of theories. Psychol. Public Policy Law.

[11] Hugenberg K, Young SG, Bernstein MJ, Sacco DF (2010). The categorization-individuation model: An integrative account of the other-race recognition deficit. Psychol. Rev..

[12] Stahl J, Wiese H, Schweinberger SR (2010). Learning task affects ERP-correlates of the own-race bias, but not recognition memory performance. Neuropsychologia.

[13] Wiese H, Kaufmann JM, Schweinberger SR (2014). The neural signature of the own-race bias: Evidence from event-related potentials. Cereb. Cortex.

[14] Herzmann G, Willenbockel V, Tanaka JW, Curran T (2011). The neural correlates of memory encoding and recognition for own-race and other-race faces. Neuropsychologia.

[15] Ito TA, Urland GR (2003). Race and gender on the brain: Electrocortical measures of attention to the race and gender of multiply categorizable individuals. J. Pers. Soc. Psychol..

[16] Ito TA, Tomelleri S (2017). Seeing is not stereotyping: The functional independence of categorization and stereotype activation. Soc. Cogn. Affect. Neurosci..

[17] Wiese H, Stahl J, Schweinberger SR (2009). Configural processing of other-race faces is delayed but not decreased. Biol. Psychol..

[18] Gajewski PD, Stoerig P, Falkenstein M (2008). ERP–correlates of response selection in a response conflict paradigm. Brain Res..

[19] Horry R, Wright DB, Tredoux CG (2010). Recognition and context memory for faces from own and other ethnic groups: A remember-know investigation. M&C.

[20] Marcon JL, Susa KJ, Meissner CA (2009). Assessing the influence of recollection and familiarity in memory for own-versus other-race faces. Psychon. Bull. Rev..

[21] Bentin S, Allison T, Puce A, Perez E, McCarthy G (1996). Electrophysiological studies of face perception in humans. J. Cogn. Neurosci..

[22] Eimer M (2000). Event-related brain potentials distinguish processing stages involved in face perception and recognition. Clin. Neurophysiol..

[23] Ofan RH, Rubin N, Amodio DM (2011). Seeing race: N170 responses to race and their relation to automatic racial attitudes and controlled processing. J. Cogn. Neurosci..

[24] Vizioli L, Rousselet GA, Caldara R (2010). Neural repetition suppression to identity is abolished by other-race faces. Proc. Natl. Acad. Sci. U. S. A..

[25] Vizioli L, Foreman K, Rousselet GA, Caldara R (2010). Inverting faces elicits sensitivity to race on the N170 component: A cross-cultural study. J. Vis..

[26] Amodio DM (2010). Coordinated roles of motivation and perception in the regulation of intergroup responses: Frontal cortical asymmetry effects on the P2 event-related potential and behavior. J. Cogn. Neurosci..

[27] Wiese H (2013). Do neural correlates of face expertise vary with task demands? Event-related potential correlates of own- and other-race face inversion. Front. Hum. Neurosci..

[28] Lv J, Yan T, Tao L, Zhao L (2015). The role of configural processing in face classification by race: An ERP study. Front. Hum. Neurosci..

[29] Sun G, Zhang G, Yang Y, Bentin S, Zhao L (2014). Mapping the time course of other-race face classification advantage: A cross-race ERP study. Brain Topogr..

[30] Senholzi KB, Ito TA (2013). Structural face encoding: How task affects the N170's sensitivity to race. Soc. Cogn. Affect. Neurosci..

[31] James MS, Johnstone SJ, Hayward WG (2001). Event-related potentials, configural encoding, and feature-based encoding in face recognition. J. Psychophysiol..

[32] Schendan HE, Ganis G, Kutas M (1998). Neurophysiological evidence for visual perceptual categorization of words and faces within 150 ms. Psychophysiology.

[33] Herrmann MJ, Ehlis AC, Ellgring H, Fallgatter AJ (2005). Early stages (P100) of face perception in humans as measured with event-related potentials (ERPs). J. Neural Transm. (Vienna).

[34] Heinze HJ (1994). Combined spatial and temporal imaging of brain activity during visual selective attention in humans. Nature.

[35] Colombatto C, McCarthy G (2017). The effects of face inversion and face race on the P100 ERP. J. Cogn. Neurosci..

[36] Wang H, Qiu R, Li W, Li S, Fu S (2020). Cultural differences in the time course of configural and featural processing for own-race faces. Neuroscience.

[37] Luck SJ, Hillyard SA (1994). Electrophysiological correlates of feature analysis during visual search. Psychophysiology.

[38] Dickter CL, Kittel JA (2012). The effect of stereotypical primes on the neural processing of racially ambiguous faces. Soc. Neurosci..

[39] Ito TA, Urland GR (2005). The influence of processing objectives on the perception of faces: an ERP study of race and gender perception. Cogn. Affect. Behav. Neurosci..

[40] Ito TA, Bartholow BD (2009). The neural correlates of race. Trends Cogn. Sci..

[41] Willadsen-Jensen EC, Ito TA (2006). Ambiguity and the timecourse of racial perception. Soc. Cogn..

[42] Willadsen-Jensen EC, Ito TA (2008). A foot in both worlds: Asian Americans' perceptions of Asian, white, and racially ambiguous faces. Group Process Intergroup Relat..

[43] Kubota JT, Ito TA (2007). Multiple cues in social perception: The time course of processing race and facial expression. J. Exp. Soc. Psychol..

[44] Dickter CL, Bartholow BD (2007). Racial ingroup and outgroup attention biases revealed by event-related brain potentials. Soc. Cogn. Affect. Neurosci..

[45] Volpert-Esmond HI, Bartholow BD (2019). Explicit categorization goals affect attention-related processing of race and gender during person construal. J. Exp. Soc. Psychol..

[46] Volpert-Esmond HI, Merkle EC, Bartholow BD (2017). The iterative nature of person construal: Evidence from event-related potentials. Soc. Cogn. Affect. Neurosci..

[47] Ito TA, Thompson E, Cacioppo JT (2004). Tracking the timecourse of social perception: The effects of racial cues on event-related brain potentials. Pers. Soc. Psychol. Bull..

[48] He Y, Johnson MK, Dovidio JF, McCarthy G (2009). The relation between race-related implicit associations and scalp-recorded neural activity evoked by faces from different races. Soc. Neurosci..

[49] Johnson R (1986). A triarchic model of P300 amplitude. Psychophysiology.

[50] Nieuwenhuis S, Aston-Jones G, Cohen JD (2005). Decision making, the P3, and the locus coeruleus-norepinephrine system. Psychol. Bull..

[51] Mercure E, Dick F, Johnson MH (2008). Featural and configural face processing differentially modulate ERP components. Brain Res..

[52] Johnson JS, Olshausen BA (2005). The earliest EEG signatures of object recognition in a cued-target task are postsensory. J. Vis..

[53] Minear M, Park DC (2004). A lifespan database of adult facial stimuli. Behav. Res. Methods Instrum. Comput..

[54] Delorme A, Makeig S (2004). EEGLAB: An open source toolbox for analysis of single-trial EEG dynamics including independent component analysis. J. Neurosci. Methods.

[55] Pion-Tonachini L, Kreutz-Delgado K, Makeig S (2019). ICLabel: An automated electroencephalographic independent component classifier, dataset, and website. Neuroimage.

[56] Luck SJ, Kappenman ES (2011). The Oxford Handbook of Event-Related Potential Components.

[57] Walker PM, Silvert L, Hewstone M, Nobre AC (2008). Social contact and other-race face processing in the human brain. Soc. Cogn. Affect. Neurosci..

[58] Faul, F., Erdfelder, E. J. B., FRG: Bonn University, Department of Psychology. GPOWER: A priori, post-hoc, and compromise power analyses for MS-DOS [Computer program]. (1992).

[59] Lakens D (2022). Sample size justification. Collabra Psychol..

[60] Rousselet GA, Husk JS, Bennett PJ, Sekuler AB (2008). Time course and robustness of ERP object and face differences. J. Vis..

[61] Brebner JL, Krigolson O, Handy TC, Quadflieg S, Turk DJ (2011). The importance of skin color and facial structure in perceiving and remembering others: An electrophysiological study. Brain Res..

[62] Rossion B (2000). The N170 occipito-temporal component is delayed and enhanced to inverted faces but not to inverted objects: An electrophysiological account of face-specific processes in the human brain. NeuroReport.

[63] Joyce C, Rossion B (2005). The face-sensitive N170 and VPP components manifest the same brain processes: The effect of reference electrode site. Clin. Neurophysiol..

[64] van Dinteren R, Arns M, Jongsma ML, Kessels RP (2014). P300 development across the lifespan: A systematic review and meta-analysis. PLoS ONE.

[65] Meissner CA, Brigham JC (2001). Thirty years of investigating the own-race bias in memory for faces: A meta-analytic review. Psychol. Public Policy Law.

[66] Button KS (2013). Power failure: Why small sample size undermines the reliability of neuroscience. Nat. Rev. Neurosci..

[67] Ioannidis JP (2008). Why most discovered true associations are inflated. Epidemiology.

[68] Szucs D, Ioannidis JP (2017). Empirical assessment of published effect sizes and power in the recent cognitive neuroscience and psychology literature. PLoS Biol..

[69] O'Toole AJ, Deffenbacher KA, Valentin D, Abdi H (1994). Structural aspects of face recognition and the other-race effect. Mem. Cognit..

